# Targeting SRC/STAT3 Signaling Impairs Cancer Stem Cell Activity by Downregulation of Hexokinase-2 in Radioresistant Triple-Negative Breast Cancer Cells

**DOI:** 10.32604/or.2026.075190

**Published:** 2026-04-22

**Authors:** Yu-Hao Huang, Yu-Ci Tu, Peng-Ju Chien, An-Jie Lee, Chia-Liang Lin, Shao-Ti Li, Yueh-Chun Lee, Wen-Wei Chang

**Affiliations:** 1 Department of Biomedical Sciences, Chung Shan Medical University, Taichung, Taiwan; 2 Division of Thoracic Surgery, Department of Surgery, Changhua Christian Hospital, Changhua, Taiwan; 3 Department of Biochemistry, School of Medicine, Chung Shan Medical University, Taichung, Taiwan; 4 Department of Radiation Oncology, Chung Shan Medical University Hospital, Taichung, Taiwan; 5 School of Medicine, Chung Shan Medical University, Taichung, Taiwan; 6 Department of Medical Research, Chung Shan Medical University Hospital, Taichung, Taiwan

**Keywords:** Triple-negative breast cancer, radioresistance, cancer stem cells, SRC, signal transducer and activator of transcription 3 (STAT3), Hexokinase-2 (HK2)

## Abstract

**Background:**

Triple-negative breast cancer (TNBC) is an aggressive subtype with poor prognosis and resistance to conventional therapies, including radiotherapy. Cancer stem cells (CSCs) drive tumor initiation, metastasis, and therapy resistance in TNBC. Identifying pathways sustaining CSCs in radioresistant TNBC is key for targeted therapies. This study examines SRC proto-oncogene (SRC) and the signal transducer and activator of transcription 3 (STAT3) activation in radioresistance and CSC maintenance.

**Methods:**

A radioresistant MDA-MB-231 TNBC cell line (231RR) was developed and compared to the parental line for CSC activity and self-renewal. Western blotting assessed molecular changes; functional assays followed SRC and STAT3 inhibitor treatment. SRCY^530F^ overexpression and hexokinase-2 (HK2) knockdown evaluated roles in CSC activity and signaling. Pathways were analyzed via metabolic assays, The Cancer Genome Atlas (TCGA) breast cancer datasets, and Harmonizome gene sets.

**Results:**

231RR cells exhibited enhanced CSC traits and upregulated SRC/STAT3 signaling, with heightened sensitivity to SRC/STAT3 inhibitors. Forced expression of SRCY530F in parental cells boosted STAT3 activation and CSC activity. SRC/STAT3 inhibition reduced HK2 without impairing glycolysis. HK2 knockdown decreased MYC proto-oncogene (c-MYC) and octamer-binding transcription factor-4 (OCT4). Finally, the suppression of epidermal growth factor receptor (EGFR) activation by gefitinib resulted in the inhibition of the SRC/STAT3/HK2 axis. TCGA data linked SRC to glycolytic signatures in breast cancer.

**Conclusions:**

The EGFR/SRC/STAT3/HK2 axis drives radioresistance and CSC maintenance in TNBC via HK2 upregulation. HK2 promotes stemness mainly through non-metabolic means, not broad metabolic shifts. Targeting this pathway could overcome radioresistance and enhance TNBC outcomes.

## 1 Introduction

Breast cancer is one of the most common malignancies in women, with 2.3 million new cases and 670,000 deaths globally in 2022 [1]. Triple-negative breast cancer (TNBC) is a highly aggressive subtype characterized by high invasiveness, metastatic potential, and poor prognosis [2,3]. Since this cancer lacks estrogen receptor (ER), progesterone receptor (PR), and erb-b2 receptor tyrosine kinase 2 (HER2) expression, treatment relies exclusively on surgery, cytotoxic chemotherapy, and radiotherapy [4].

Radiotherapy is a key component of TNBC treatment, but the development of radioresistance frequently results in treatment failure and higher recurrence rates compared with other breast cancer subtypes [5,6]. Cancer stem cells (CSCs) have an important role in the development of radioresistance, recurrence, and metastasis in TNBC. CSCs are a subpopulation of tumor cells with self-renewal capacity and resistance to therapy [7–9]. The SRC Proto-Oncogene (SRC), a non-receptor tyrosine kinase, has an important role in the development of CSC maintenance and therapeutic resistance [10]. The activation of signal transducer and activator of transcription 3 (STAT3) by SRC has been demonstrated to play a critical role in maintaining CSC phenotypes across various cancers [11,12]. Inhibition of SRC, for example, with dasatinib, has been shown to impair DNA repair and reduce CSC activity [13,14]. However, how this SRC/STAT3 pathway contributes specifically to radioresistance in TNBC remains poorly understood.

CSCs in aggressive tumors, including TNBC, often depend on aerobic glycolysis (the Warburg effect) and biosynthetic precursors to generate energy even under oxygen-rich conditions [15,16]. Hexokinase-2 (HK2), the main rate-limiting enzyme in glycolysis, is frequently upregulated in malignancies [17,18], promoting metabolic reprogramming, enhanced cell survival, and therapy resistance [15,19,20]. HK2 also has non-metabolic functions, including nuclear translocation, where it regulates stemness-related gene expression and supports CSC maintenance [21].

In prior work, we established a radioresistant MDA-MB-231 subline (231RR) with enhanced CSC properties via upregulated Notch1 and β-catenin signaling [22,23]. In this study, we aimed to investigate SRC/STAT3 activation in radioresistant TNBC and its link to CSC maintenance.

## 2 Materials and Methods

### 2.1 Cell Line and Culture Conditions

Human TNBC cell lines, including MDA-MB-231 (cat. No. 60425; referred to as 231-P in this study) and Hs578T (cat. No. 60120), were obtained from the Bioresource Collection and Research Center (Hsinchu, Taiwan) and cultured in 6-cm dishes containing Dulbecco’s Modified Eagle’s Medium (DMEM) (cat. No. 11965092, Gibco^™^, Thermo Fisher Scientific, Waltham, MA, USA) supplemented with 10% fetal bovine serum (FBS) (cat. No. SH30071, HyClone, Logan, UT, USA), 1 mM sodium pyruvate (cat. No. 11360070, Gibco^™^, Thermo Fisher Scientific), 2 mM L-glutamine (cat. No. 25030081, Gibco^™^, Thermo Fisher Scientific), and 100 µg/mL of antibiotics (penicillin/streptomycin/amphotericin B; cat. No. 03-033-1B, Biological Industries, Beit-Haemek, Israel), in accordance with ATCC guidelines. The authenticity of all TNBC cell lines was verified by short tandem repeat (STR) profiling performed at the Center for Genomic Medicine, National Cheng Kung University (Tainan, Taiwan). All cell lines were confirmed to be free of mycoplasma contamination using the MycoAlert^™^ PLUS Mycoplasma Detection Kit (cat. No. LT07-710, Lonza Bioscience, Walkersville, MD, USA). Radioresistant MDA-MB-231 cells (designated as 231RR) were generated as previously described (15). To maintain their radioresistant phenotype, 231RR cells were exposed weekly to 2 Gy of ionizing radiation using an Elekta Versa HD linear accelerator (Elekta, Stockholm, Sweden).

### 2.2 Chemicals and Antibodies

Dasatinib (cat. No. 11498), C188-9 (cat. No. 30928) and Gefitinib (cat. No. 13166) were purchased form Cayman Chemical (Michigan, USA) and dissolved in dimethyl sulfoxide (DMSO, cat. No. 67-68-5, MilliporeSigma, Burlington, MA, USA). Primary antibodies specific to glucose transporter-1 (GLUT1) (cat. No. A6982, 1:1000), gamma histone variant H2AX (γ-H2AX) (cat. No. AP0687, 1:500), HK1 (cat. No. A0533, 1:1000), HK2 (cat. No. A20829, 1:1000), p-epidermal growth factor receptor (EGFR)Y1173 (cat. No. AP0992, 1:500), lactate dehydrogenase (LDHA) (cat. No. A0861, 1:1000), p-SRCY416 (cat. No. AP0480, 1:500), SOX2 (cat. No. A0561, 1:500), SRC (cat. No. A19119, 1:1000), pyruvate dehydrogenase E1 subunit alpha 1 (PDHA1) (cat. No. A13687, 1:1000), and pyruvate kinase isozymes M1/M2 (PKM1/2) (cat. No. A13905, 1:1000) were purchased from ABclonal Inc. (Woburn, MA, USA). Primary antibodies specific to EGFR (cat. No. GTX121919, 1:1000), p-STAT3Y705 (cat. No. GTX118000, 1:1000), STAT3 (cat. No. GTX104616, 1:1000), GAPDH (cat. No. GTX100118, 1:5000), and Tubulin (cat. No. GTX27291, 1:2000) were purchased from GeneTex International Corporation (Hsinchu City, Taiwan). Primary antibodies specific to c-MYC (cat. No. 10828-1-AP, 1:1000) and OCT4 (cat. No. 11263-1-AP, 1:1000) were purchased from Proteintech Group Inc. (Rosemont, IL, USA). Peroxidase-conjugated secondary anti-rabbit IgG (cat. No. 31460, 1:10,000) was purchased from Thermo Fisher Scientific Inc. (Waltham, MA, USA). GAPDH was used as an internal control.

### 2.3 Clonogenic Assay

231-RR and Hs578T cells were seeded at a density of 200 cells per well in 12-well plates and incubated at 37°C for 7–10 days to allow colony formation. Colonies were fixed with 2% formaldehyde at room temperature for 5 min and subsequently stained with 1% crystal violet (cat. No. 32675, MilliporeSigma) for 1 h at room temperature. Colonies containing ≥ 50 cells were imaged using an inverted light microscope (AE30; Motic Incorporation, Ltd., Hong Kong, China) and manually counted.

### 2.4 Tumorsphere Cultivation

Tumorsphere formation assays were performed as previously described [24]. Briefly, single-cell suspensions of MDA-MB-231, 231RR (radioresistant subline derived from MDA-MB-231), and Hs578T cells were cultured in DMEM/F12 medium (cat. No. 11320033, Gibco^™^, Thermo Fisher Scientific) supplemented with 0.4% BSA (cat. No. A3311, MilliporeSigma), 1× serum-free B27 supplement (cat. No. 17504-044, Gibco^™^, Thermo Fisher Scientific), 20 ng/mL EGF (cat. No. AF-100-15, PeproTech, Cranbury, NJ, USA), 20 ng/mL basic FGF (cat. No. 100-18B, PeproTech), 5 μg/mL insulin (cat. No. I5500, MilliporeSigma), 1 μg/mL hydrocortisone (cat. No. H0135, MilliporeSigma), and 4 μg/mL heparin (cat. No. H0878, MilliporeSigma). Cells (1 × 10^4^ per well) were seeded into ultra-low attachment 6-well plates (Greiner Bio-One, Kremsmünster, Austria) and incubated at 37°C in a humidified atmosphere containing 5% CO_2_ for 7–10 days to allow tumorsphere formation. Tumorspheres were defined as spherical cellular aggregates with a diameter greater than 50 μm.

### 2.5 Western Blotting

Whole cell lysates of MDA-MB-231, 231-RR, and Hs578T cells were prepared by cell lysis in RIPA buffer (25 mM Tris-HCl pH 7.6, 150 mM NaCl, 1% NP-40, 1% sodium deoxycholate and 0.1% SDS). Protein concentration was determined using the BCA Protein Assay Kit (cat. No. A55865, Thermo Fisher Scientific). 25 μg of total proteins per lane were loaded into 10% SDS-PAGE gel for protein separation by electrophoresis. Subsequently, the separated proteins were transferred onto 0.45 μm PVDF membranes (cat. no. 66547; Pall Corporation, Washington, NY, USA). After blocking with nonfat dry milk 5% w/v in TBS buffer at room temperature for 1 h, the membranes were then incubated with primary antibodies at 4°C for 16 h. After washing with 0.1% Tween-20/TBS buffer, the membranes were incubated with peroxidase-conjugated secondary antibody at room temperature for 1 h. The signal was then developed using Pierce ECL Western Blotting Substrate (cat. No. 32106, Thermo Fisher Scientific) and imaged using the Amersham Imager 680 (Cytiva, Marlborough, MA, USA). Band intensities were quantified using ImageJ software (version 1.54k, National Institutes of Health, Bethesda, MD, USA) for densitometric analysis. Protein expression levels were normalized to GAPDH as the loading control. The dilution ratios for primary and secondary antibodies are described in Section 2.2.

### 2.6 Cell Mito Stress Test

MDA-MB-231 and 231-RR cells were seeded into Seahorse XF96 Cell Culture Microplates (cat. No. 103794-100, Agilent Technologies, Santa Clara, CA, USA) at a density of 1 × 10^3^ cells per well and cultured until reaching 80%–90% confluence. Prior to the Seahorse XF Cell Mito Stress Test (cat. No. 103015-100, Agilent Technologies), the sensor cartridge was hydrated in Seahorse XF Calibrant (cat. No. 103059-000, Agilent Technologies) according to the manufacturer’s instructions. On the day of the assay, cells were washed once and incubated in XF Seahorse Base Medium DMEM (cat. No. 103575-100, Agilent Technologies) supplemented with 5.5 mM glucose, 1 mM sodium pyruvate, 4 mM L-glutamine, and 1× non-essential amino acids (cat. No. 11140050, Gibco, ThermoFisher Scientific) to mimic both cell growth. The assay medium was prepared according to the manufacturer’s protocol. Cells were then equilibrated in a non-CO_2_ incubator at 37°C for 1 h prior to measurement. Oxygen consumption rate (OCR) and extracellular acidification rate (ECAR) were measured using the Seahorse XF96 Analyzer (model No. S7800A, Agilent Technologies) under basal conditions and following sequential injections of mitochondrial inhibitors provided by the Seahorse XF Cell Mito Stress Test Kit: oligomycin (1 µM), carbonyl cyanide 4-(trifluoromethoxy)phenylhydrazone (FCCP) (1 µM), and rotenone/antimycin A (0.5 µM each). Data were analyzed using the Seahorse XFe96 Extracellular Flux Analyzer (Agilent Technologies). Each condition was performed in three replicate wells per cell line.

### 2.7 Gene Set Enrichment Analysis (GSEA)

To investigate the potential association between SRC expression and specific metabolic pathways, RNA-seq transcriptomic data from 1212 breast cancer (BRCA) patients were obtained from The Cancer Genome Atlas (TCGA; https://portal.gdc.cancer.gov). Patients were stratified into two cohorts based on SRC mRNA abundance using the median RNA-Seq by Expectation-Maximization (RSEM) normalized expression value as the cutoff: an SRC-high group (*n* = 606) and an SRC-low group (*n* = 606). Gene Set Enrichment Analysis (GSEA) was subsequently performed using GSEA software (version 4.3.2) to identify biological pathways significantly enriched in the SRC-high phenotype. The analysis focused on glycolysis-related signatures from the Molecular Signatures Database (MSigDB, version 2025.1.Hs), including the “HALLMARK_GLYCOLYSIS” and “REACTOME_GLYCOLYSIS” gene sets. Statistical significance was assessed by calculating the Normalized Enrichment Score (NES), nominal *p*-value, and False Discovery Rate (FDR). Gene sets with a *p*-value < 0.05 and FDR < 0.25 were considered statistically significant.

### 2.8 Lentiviral shRNA-Mediated Knockdown of HK2

The packaging plasmids pCMVΔ8.91 and pMD.G, along with gene-specific shRNAs targeting HK2 (#1, TRCN0000037669; #2, TRCN0000037672) and LacZ (control, TRCN0000231722), were obtained from the National RNAi Core Facility (Academia Sinica, Taipei, Taiwan). Lentiviral particles were generated by co-transfecting HEK-293T cells with a plasmid mixture containing shRNA (2.5 μg), pCMVΔ8.91 (2.25 μg), and pMD.G (0.25 μg) using NTRII DNA transfection reagent (cat. No. JT97-N002M; T-Pro Biotechnology, New Taipei City, Taiwan). Viral supernatants were harvested 48 h post-transfection, filtered through a 0.45 μm filter. For transduction, 231RR or Hs578T cells were seeded into 3.5 cm culture dishes at a density of 1 × 10^5^ cells/dish, and the shRNA-carrying lentivirus was added at a multiplicity of infection (MOI) of 1 in the presence of 8 μg/mL polybrene (cat. No. TR-1003-G, Sigma-Aldrich, St Louis, MO, USA). After 24 h of infection, the medium was replaced with fresh culture medium supplemented with 2 μg/mL puromycin to select for stably transduced cells.

### 2.9 Gene Signature and Correlation Analysis

Gene signatures for EGFR, SRC, and STAT3 were extracted from the Hub Proteins Protein-Protein Interactions dataset within the Harmonizome database (version 3.0, released on Nov. 20, 2024; available at https://maayanlab.cloud/Harmonizome/) [25]. Genes included in each signature were selected based on protein-protein interaction data curated from the database, including all documented interacting partners for each hub protein (EGFR, SRC, and STAT3). To investigate the relationships between these signaling pathways and metabolic markers, correlation analyses were conducted using the Gene Expression Profiling Interactive Analysis 2 (GEPIA2) platform [26] with data from The Cancer Genome Atlas (TCGA) breast cancer specimens. Specifically, pairwise correlations were analyzed between HK2 expression levels and the SRC gene signature, between the EGFR and STAT3 gene signatures, and between the EGFR gene signature and HK2 expression. Correlation analyses were performed using Pearson’s correlation coefficient through the GEPIA2 platform, with statistical significance set at *p* < 0.05. As these were hypothesis-driven pairwise correlations, multiple testing correction was not applied.

### 2.10 Statistical Analysis

Data from MTT assay, tumorsphere assays and OCR/ECAR analyses were presented as mean ± standard deviation (SD). Statistical analyses were performed using GraphPad Prism 5.0 (GraphPad Software, San Diego, CA, USA). Comparisons between two groups were conducted using the unpaired Student’s *t*-test. Overall survival (OS) was analyzed using the Kaplan–Meier method, and differences between groups were evaluated with the log-rank test. A *p*-value < 0.05 was considered statistically significant.

## 3 Results

### 3.1 Activation of SRC/STAT3 Supports Cell Growth in Radioresistant TNBC Cells

**Figure 1:**
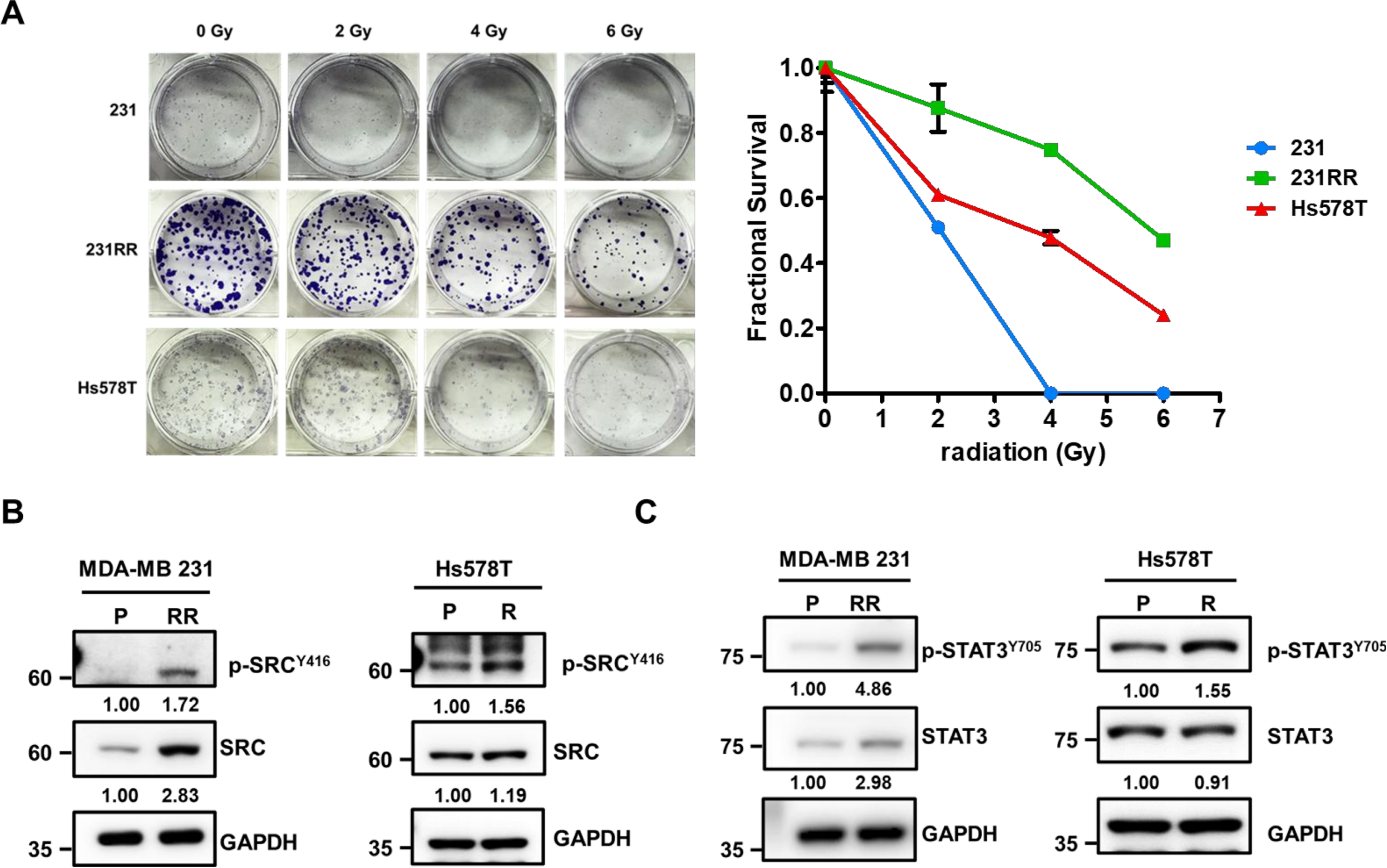
Activation of the SRC proto-oncogene (SRC)/the signal transducer and activator of transcription 3 (STAT3) pathway in radioresistant triple negative breast cancer (TNBC) cells. (**A**) A clonogenic survival assay was performed to compare the responses of parental MDA-MB-231 (231), radioresistant MDA-MB-231 (231RR), and Hs578T cells to ionizing radiation (0–6 Gy). Representative images of crystal violet-stained colonies are shown (left panel), and quantitative analysis of fractional survival is presented (right panel) as mean ± SD. (**B**,**C**) Western blot analysis of p-SRC^Y416^, SRC, p-STAT3^Y705^, and STAT3 in TNBC cells of the following types: parental (P), radioresistant (RR), or exposed to 6 Gy of ionizing radiation (R). GAPDH was used as a loading control. Numbers below the blots indicate relative expression levels normalized to parental cells. Molecular weights are indicated in kDa.

To establish the radioresistant phenotype, MDA-MB-231 cells were subjected to repeated exposures of 2 Gy ionizing radiation, reaching a cumulative dose of 32 Gy, and subsequently designated as 231RR cells. In parallel, our previous findings identified the Hs578T cell line as intrinsically more resistant to radiation [27]. he radioresistant phenotype of both 231RR and Hs578T cells was validated through clonogenic survival assays. Compared to parental MDA-MB-231 cells (231 cells), both 231RR and Hs578T cells displayed better capability to form colonies under irradiation treatment. At a 6 Gy radiation dose, the 231RR and Hs578T cells maintained substantial colony-forming capacity, whereas the colony formation in the 231 cells was completely abolished (Fig. 1A). Protein analysis revealed that the phosphorylation levels of SRC and STAT3 were elevated in the radioresistant 231RR cells compared to their parental 231 counterparts (Fig. 1B,C, left panels). Furthermore, Hs578T cells that received a single exposure to 2 Gy of ionizing radiation exhibited increased phosphorylation of SRC and STAT3 (see Fig. 1B,C, right panels). These results imply that the radiation-induced activation of the SRC/STAT3 pathways is a conserved phenomenon across different TNBC cell lines.

**Figure 2:**
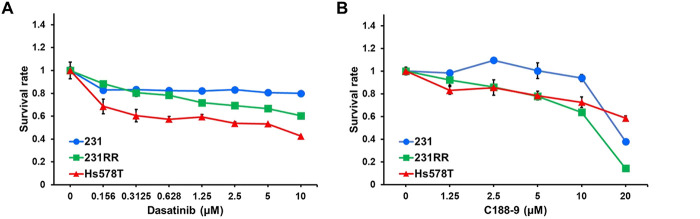

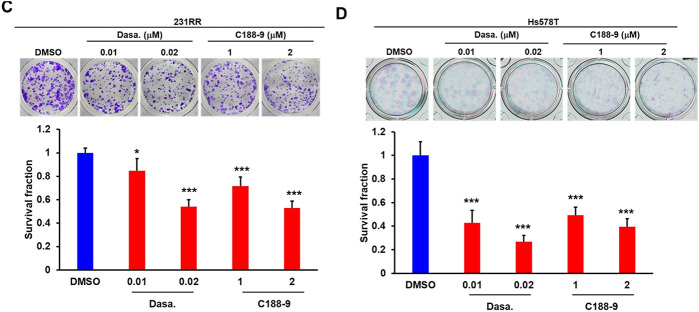
Radioresistant TNBC cells are sensitive to SRC/STAT3 inhibition. (**A**,**B**) MDA-MB-231 TNBC cells, radioresistant MDA-MB-231 (231RR) cells and Hs578T cells were treated with increasing concentrations of dasatinib (0–10 µM, A) or C188-9 (0–20 µM, B) for 72 h, after which cell viability was evaluated using an MTT assay. (**C**,**D**) 231RR and Hs578T cells (200 cells/well) were seeded in 12-well plates and treated with dasatinib (**C**) or C188-9 (**D**), respectively. Colonies were stained with crystal violet and counted 10 days after seeding. *p < 0.05; ***p < 0.001.

To determine the functional contributions of SRC and STAT3 signaling in radioresistant TNBC cells, we used the pharmacological inhibitors: dasatinib for SRC and C188-9 for STAT3. Inhibiting SRC/STAT3 signaling resulted in more pronounced suppression of cell proliferation in 231RR and Hs578T cells than in 231 cells (Fig. 2A,B). Furthermore, blocking SRC or STAT3 activity significantly reduced the clonogenic potential of 231RR and Hs578T cells in long-term colony formation assays (Fig. 2C,D), highlighting the essential role of SRC/STAT3 signaling in maintaining the radioresistant phenotype.

Phosphorylation of the histone variant H2AX at serine 139, which leads to the formation of γH2AX, is a well-established, sensitive early cellular marker of DNA double-strand breaks caused by ionizing radiation [28]. In 231RR cells, the pharmacological inhibition of SRC and STAT3 increased the accumulation of γH2AX following radiation exposure (Fig. S1A), which was further confirmed by immunofluorescence analysis showing significantly elevated γH2AX foci (Fig. S1D,E). These results indicate that the suppression of these signaling pathways potentiates radiation-induced DNA damage. Moreover, we successfully produced 231 cells that stably expressed a constitutively active SRC by transfecting the SRC^Y530F^ mutant [29]. SRC hyperactivation enhanced STAT3 phosphorylation (Fig. S1B), indicating that STAT3 acts downstream of SRC signaling. Additionally, a significant decrease in γH2AX accumulation following radiation exposure was observed in the 231 cells (Fig. S1C). Taken together, these results suggest that blocking SRC/STAT3 enhances radiation-induced DNA damage and impairs the proliferative capacity of radioresistant TNBC cells.

### 3.2 Blockade of SRC/STAT3 Inhibits Cancer Stemness in Radioresistant TNBC Cells

**Figure 3:**
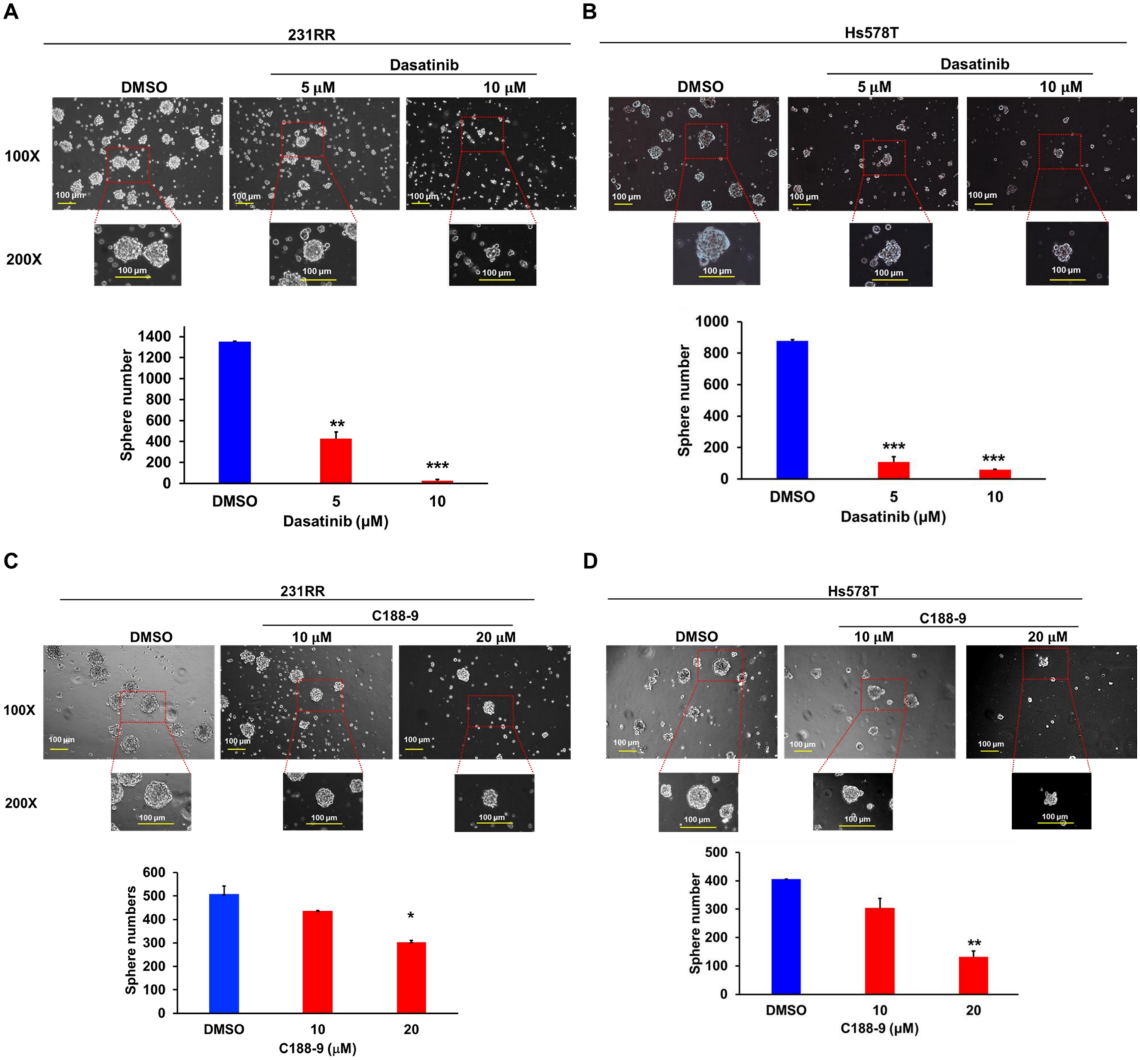
Pharmacological inhibition of SRC/STAT3 activity impairs CSC activity in radioresistant TNBC cells. 231RR (**A**,**C**) or Hs578T (**B**,**D**) cells were treated with dasatinib (**A**,**B**) or C188-9 (**C**,**D**), after which CSC activity was assessed using the tumorsphere formation assay. Representative tumorsphere images are shown at 100× magnification (upper panels) with corresponding 200× magnification images (middle panels) indicating the regions marked by red dotted lines. Quantitative analyses are presented in the lower panels as the mean ± SD. *, *p* < 0.05; **, *p* < 0.01; ***, *p* < 0.001. Scale bars represent 100 µm in length.

In our previous studies, we demonstrated that 231RR cells exhibit significantly higher CSC activity compared to their parental 231 counterparts [23,27]. A previous report has also indicated that pharmacological inhibition of SRC signaling effectively suppresses the functional activity of patient-derived gastric CSCs [30]. These findings suggest that SRC has a vital role in regulating CSC-associated phenotypes in cancers. We further investigated whether the SRC/STAT3 signaling axis plays a critical role in sustaining CSC activity in radioresistant TNBC cells. Pharmacological inhibition of SRC or STAT3 using dasatinib or C188-9, respectively, significantly reduced tumorsphere-forming capacity in both 231RR and Hs578T cells (Fig. 3A–D).

**Figure 4:**
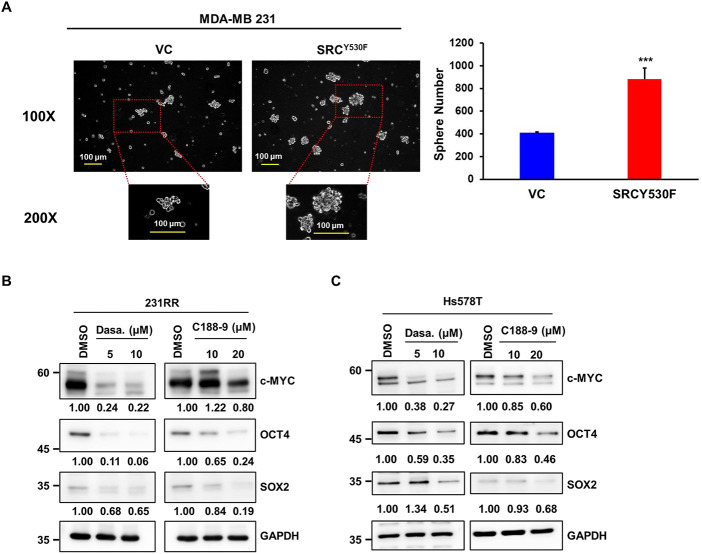
Suppression of SRC/STAT3 activity reduces the levels of cancer stemness factors. (**A**) MDA-MB-231 cells were transduced with lentiviruses carrying an empty vector (VC) or a constitutively active SRC mutant (SRC^Y530F^). CSC activity was assessed using the tumorsphere formation assay. Representative tumorsphere images are shown at 100× magnification (upper panels) with corresponding 200× magnification images (lower panels) indicating the regions marked by red dotted lines. Quantitative analysis is presented in the right panel as mean ± SD. ***p < 0.001. Scale bars represent 100 µm. (**B**,**C**) 231RR (**B**) and Hs578T (**C**) cells were treated with dasatinib or C188-9 for 48 h, after which total proteins were collected. MYC proto-oncogene (c-MYC), octamer-binding transcription factor 4 (OCT4), and SRY-box transcription factor 2 (SOX2) expression levels were analyzed by Western blot. GAPDH was used as a loading control. Numbers below the blots indicate relative expression levels normalized to DMSO control. Molecular weights are indicated in kDa.

Furthermore, enforced expression of the constitutively active mutant SRCY530F in 231 cells resulted in a significant increase in CSC activity (Fig. 4A), further supporting the promotional role of SRC in cancer stemness within TNBC cells. Mechanistically, we found that inhibiting either SRC or STAT3 resulted in significant downregulation of key pluripotency-associated transcription factors, such as c-MYC, OCT4, and SOX2, in both 231RR (Fig. 4B) and Hs578 T cells (Fig. 4C). Collectively, these results show that the SRC/STAT3 signaling pathway has a beneficial effect on CSC action in radioresistant TNBC cells and emphasize its possible function as a treatment goal.

### 3.3 HK2 Is Upregulated via SRC/STAT3 Signaling in Radioresistant TNBC Cells

**Figure 5:**
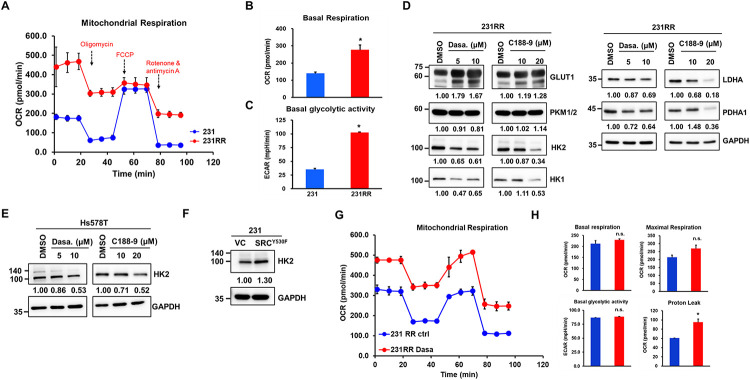
Hexokinase-2 (HK2) upregulation is mediated by SRC/STAT3 signaling in radioresistant TNBC cells. (**A**) Oxygen consumption rate (OCR) profiles of the parental (231) and radioresistant (231RR) MDA-MB-231 cell lines after the administration of metabolic compounds. (**B**,**C**) Quantification of basal OCR and maximal ECAR in 231 and 231RR cells. (**D**,**E**) Western blot analysis of glucose transporter 1 (GLUT1), pyruvate kinase M1/2 (PKM1/2), lactate dehydrogenase A (LDHA), pyruvate dehydrogenase A1 (PDHA1), HK1 and HK2 in 231RR and Hs578T cells treated with dasatinib or C188-9 for 48 h. Inserted numbers indicate relative expression levels compared to the control. (**F**) Western blot analysis of parental MDA-MB-231 (231) cells transduced with lentiviral vectors encoding an empty vector (VC) or a constitutively active SRC mutant (SRC^Y530F^). (**G**,**H**) OCR and ECAR analysis of 231RR cells treated with dasatinib (Dasa) or vehicle control (ctrl). n.s., not significant. *p < 0.05.

Previous studies have suggested that aerobic glycolysis may contribute to the development of radioresistance in various cancer types [31]. Here, we evaluated the mitochondrial function of 231 parental cells and 231RR cells by measuring their oxygen consumption rate (OCR) (Fig. 5A). An increase in both basal respiration, measured by OCR (Fig. 5B), and basal glycolytic activity, measured by ECAR (Fig. 5C), was observed in 231RR cells prior to oligomycin addition. These results indicate that 231RR cells exhibit enhanced mitochondrial respiratory activity and greater metabolic flexibility, which may contribute to their altered, therapy-resistant phenotype compared with parental 231 cells. Consistent with this, SRC has previously been identified as a key regulator of glucose metabolism in breast cancer stem cells [32]. Bioinformatic analysis of The Cancer Genome Atlas breast cancer datasets revealed that glycolytic activity is significantly higher in tumors with elevated SRC expression (Fig. S2). To investigate whether the SRC–STAT3 signaling axis regulates aerobic glycolysis in radioresistant TNBC cells, we assessed the expression of key glycolytic regulators in 231RR cells following treatment with the SRC inhibitor dasatinib or the STAT3 inhibitor C188-9. Inhibition of SRC or STAT3 significantly reduced HK2 expression in both 231RR and Hs578T cells (Fig. 5D,E). These findings further support the role of the SRC/STAT3 signaling in the metabolic reprogramming of these cells. In contrast, the enforced expression of the constitutively active SRCY530F mutant in the parental 231 cells resulted in elevated HK2 expression (Fig. 5F). Despite the reduction in HK2 expression following dasatinib treatment (Fig. 5D,E), basal respiration and basal glycolytic activity remained unchanged. In contrast, maximal respiration showed a trend toward an increase (*p* = 0.09), and proton leak was significantly elevated (Fig. 5G,H). These metabolic changes suggest that dasatinib induces mild mitochondrial uncoupling in 231RR cells.

Given the established role of HK2 in regulating glycolytic flux, these findings imply that HK2 may contribute to the radioresistant phenotype of 231RR cells through mechanisms that are at least partly independent of its metabolic function. Overall, the data indicate that SRC/STAT3-driven HK2 upregulation may play a predominantly non-metabolic role in radioresistant TNBC cells, beyond modest effects on glycolysis. Based on these findings, we decided to examine the role of HK2 in maintaining CSC activity independently of metabolic flux.

### 3.4 HK2 Maintains CSC Activity of Radioresistant TNBC Cells

**Figure 6:**
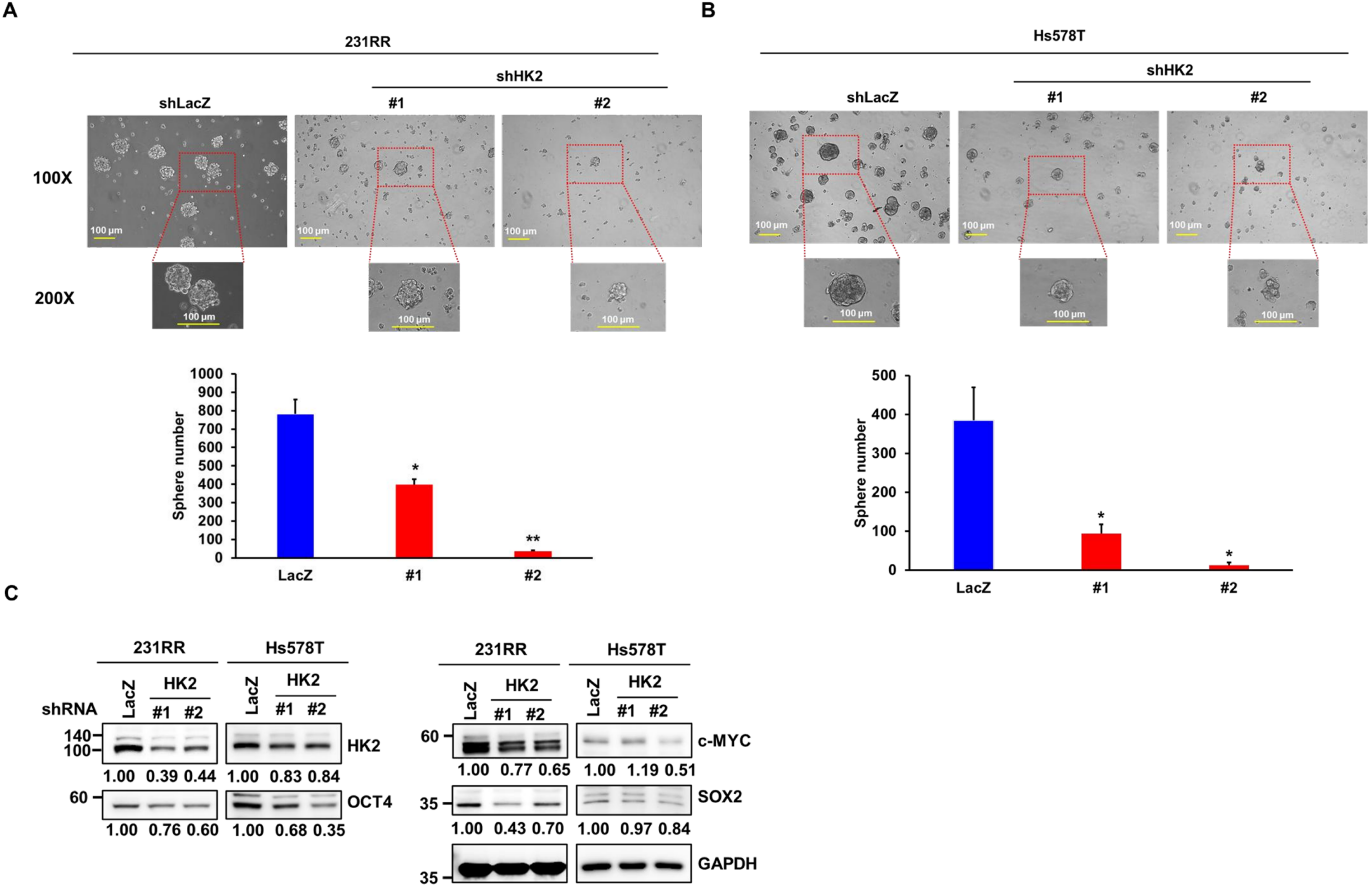
HK2 participates in breast cancer stem cell (CSC) properties. (**A**,**B**) 231RR (**A**) and Hs578T (**B**) cells were transduced with lentiviruses expressing either control (shLacZ) or HK2-targeting (shHK2#1 and shHK2#2) shRNAs. CSC activity was assessed using a tumorsphere formation assay. Representative tumorsphere images are shown at 100× magnification (upper panels) with corresponding 200× magnification images (middle panels) indicating the regions marked by red dotted lines. Quantitative analyses are presented in the lower panels as mean ± SD. *p < 0.05; **p < 0.01. Scale bars: 100 µm. (**C**) Western blot analysis of HK2, OCT4, c-MYC and SOX2 expression 72 h after transduction with shLacZ, shHK2#1 or shHK2#2 lentiviruses in 231RR or Hs578T cells. GAPDH was used as a loading control. Numbers below the blots indicate relative expression levels normalized to the control. Molecular weights are indicated in kDa.

Building on the observation that HK2 is regulated by SRC/STAT3 with limited impact on global metabolism, we examined whether HK2 sustains CSC properties through non-canonical mechanisms. Previous studies have indicated that HK2 has non-metabolic functions that contribute to tumor progression. These metabolic functions include promoting immune evasion through programmed death-ligand 1 (PD-L1) upregulation and enhancing cancer stemness by maintaining CD133 expression [33,34]. herefore, we further investigated the contribution of HK2 to the CSC properties of radioresistant TNBC cells. Silencing of HK2 via shRNA-mediated knockdown significantly impaired the tumorsphere-forming capacity in both 231RR (Fig. 6A) and Hs578T cells (Fig. 6B). Furthermore, the expression levels of the core pluripotency-associated transcription factors, including c-MYC, OCT4, and SOX2, were reduced in HK2-depleted 231RR and Hs578T cells (Fig. 6C). These results suggest that HK2 plays a vital role in maintaining the stem-like characteristics of radioresistant TNBC cells, regardless of its involvement in glycolysis.

### 3.5 EGFR Activation Modulates the SRC/STAT3/HK2 Signaling Axis in TNBC

**Figure 7:**
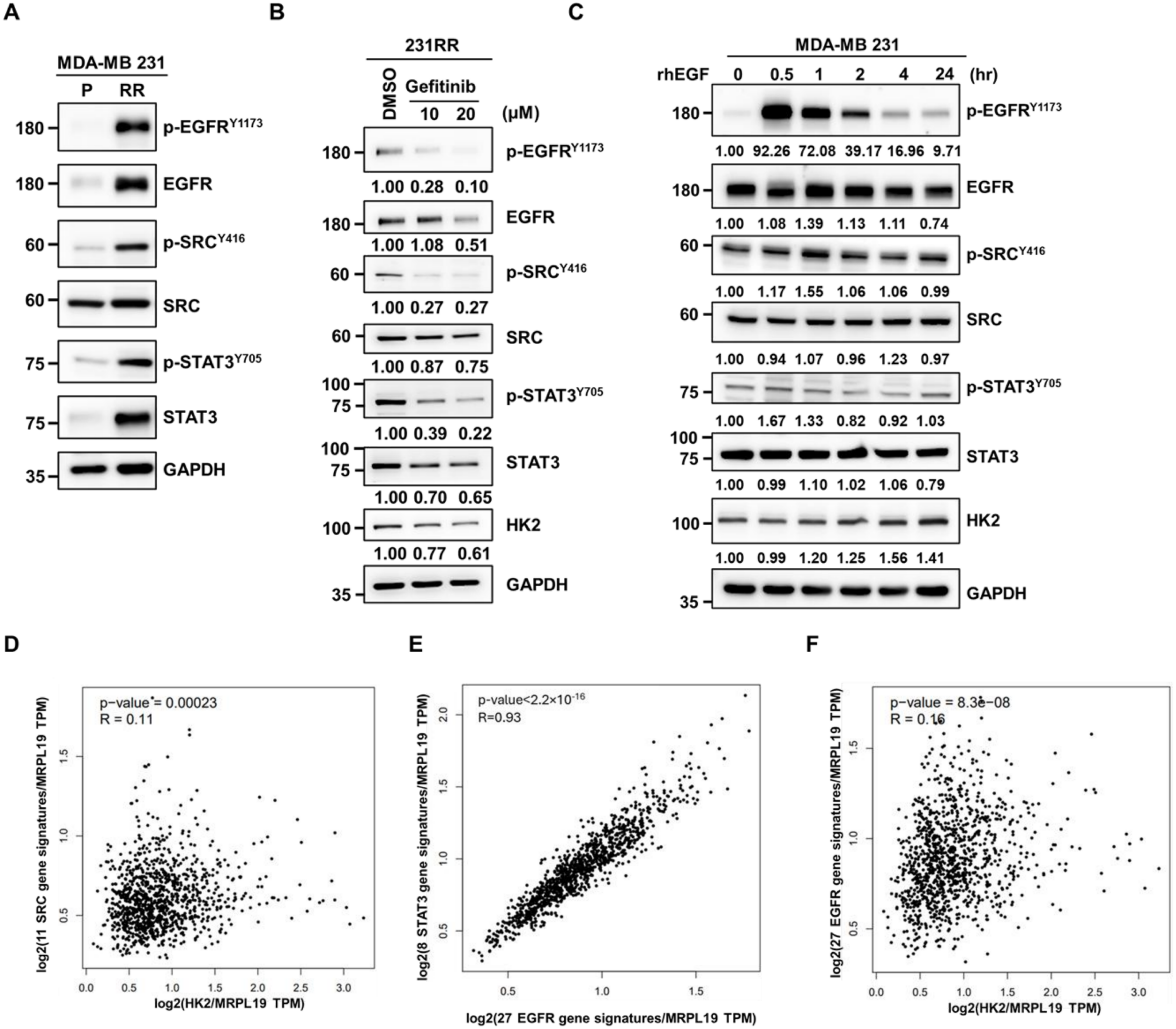
EGFR activation induces the SRC/STAT3/HK2 signaling axis in TNBC. (**A**) Western blot analysis of phosphorylated and total levels of EGFR, SRC and STAT3 in parental (231 P) and radioresistant (231 RR) MDA MB 231 cells. GAPDH was used as a protein loading control. Numbers below the blots indicate relative expression levels normalized to the control. Molecular weights are indicated in kDa. (**B**) Gefitinib was administered to 231 RR cells for 48 h, after which the levels of EGFR, p-EGFR^Y1173^, SRC, p-SRC^Y416^, STAT3, p-STAT3^Y705^ and HK2 were analyzed by Western blot. GAPDH was used as a protein loading control. Numbers below the blots indicate relative expression levels normalized to the control. Molecular weights are indicated in kDa. (**C**) Serum-starved MDA-MB-231 cells were stimulated with 40 ng/mL of recombinant human EGF (rhEGF) and harvested at the indicated time points. EGFR, p-EGFR^Y1173^, SRC, p-SRC^Y416^, STAT3, p-STAT3^Y705^ and HK2 levels were examined by Western blot. GAPDH was used as a protein loading control. Numbers below the blots indicate relative expression levels normalized to the control. Molecular weights are indicated in kDa. (**D**–**F**) Gene signatures related to EGFR, SRC and STAT3 were obtained from the Harmonizome 3.0 database [25]. Correlation analyses were performed using TCGA breast cancer specimens in the GEPIA2 platform [26] between the HK2 and SRC signature (**D**), the EGFR and STAT3 signatures (**E**), and the EGFR signature and HK2 (**F**).

EGFR has been identified as an upstream regulator of the SRC/STAT3 signaling axis in various malignancies [35]. Protein analysis showed that both total and phosphorylated EGFR levels were significantly elevated in 231RR cells compared with their parental 231 counterparts, indicating enhanced EGFR expression and activation in the radioresistant cells (Fig. 7A). The selective tyrosine kinase inhibitor gefitinib was used to pharmacologically inhibit EGFR, effectively attenuating SRC and STAT3 phosphorylation and leading to downregulation of HK2 expression in 231RR cells (Fig. 7B). Conversely, treatment with exogenous EGF activated the SRC/STAT3 pathway, leading to robust HK2 upregulation in 231 parental cells (Fig. 7C). Overall, these findings suggest that EGFR acts as a key upstream regulator of the SRC/STAT3/HK2 signaling pathway in radioresistant TNBC cells. Using the GEPIA2 analytical platform [26], we found positive correlations between HK2 mRNA and the SRC gene signature (Fig. 7D), between EGFR and the STAT3 gene signature (Fig. 7E), and between HK2 mRNA and the EGFR gene signature (Fig. 7F) in breast cancer specimens obtained from the TCGA dataset. These *in silico* results support and extend our experimental findings, further confirming the clinical relevance of the SRC/STAT3/HK2 and EGFR signaling axis in breast cancer.

## 4 Discussion

TNBC remains one of the most therapeutically challenging breast cancer subtypes due to its intrinsic heterogeneity, high relapse rate, and absence of targeted therapies [36,37]. The development of radioresistance is often driven by CSCs, which exhibit enhanced abilities to repair DNA damage, adapt to metabolic changes, and resist apoptotic signals. These properties enable CSCs to survive and proliferate despite exposure to radiation, contributing to treatment resistance and tumor recurrence [38]. Our findings indicate that the EGFR/SRC/STAT3 axis is central to maintaining CSC properties and radioresistance in TNBC, consistent with previously published evidence that RTK–STAT3–SRC signaling supports CSC survival and EGFR inhibitor resistance [39].

Glycolysis-targeted therapeutic strategies have emerged as a promising approach for suppressing tumor growth, metastatic progression, and resistance to conventional therapies. HK2, a key rate-limiting enzyme in the glycolytic cascade, has been implicated not only in metabolic regulation but also in driving resistance to endocrine therapies in breast cancer. Notably, activation of E2F transcription factors has been shown to transcriptionally upregulate HK2, thereby enhancing glycolytic flux and promoting cancer cell proliferation [40]. Similarly, the hypoxia-inducible factor 1-alpha (HIF-1α) further reinforces the glycolytic phenotype through direct transcriptional activation of HK2 and glucose transporter 1 (GLUT1). Additionally, oncogenic activation of the PI3K/AKT/mTOR pathway promotes the stabilization and transcriptional activity of HIF-1α, sustaining the expression of glycolysis-related genes and leading to elevated lactate production [41]. These convergent signaling and metabolic pathways collectively highlight HK2 as a central node in maintaining the aggressive, therapy-resistant phenotype in TNBC.

A key contribution of our study is the identification of HK2 as a downstream effector of SRC/STAT3 signaling in radioresistant TNBC. Although HK2 is traditionally considered to be a glycolytic enzyme, there is growing evidence to suggest that it has non-metabolic functions. These include stabilizing CSC surface markers such as CD133 and modulating immune evasion by upregulating PD-L1 expression [33,34]. Although pharmacological inhibition of SRC suppresses HK2 expression, our metabolic analyses revealed minimal impact on global glycolytic flux. These findings suggest that, in radioresistant TNBC cells, HK2 may predominantly function as a transcriptional co-regulator of stemness rather than as a metabolic enzyme. This concept is supported by recent findings in acute myeloid leukemia, where HK2 was shown to translocate to the nucleus and interact with chromatin remodeling complexes to modulate stem-like gene expression programs [21]. Furthermore, a recent study demonstrated that SRC-mediated phosphorylation of HK2 can alter its subcellular localization and function in glioblastoma independently of its metabolic activity [42]. Together, these findings raise the intriguing possibility that similar post-translational modifications may occur in TNBC, contributing to the non-metabolic, pro-stemness functions of HK2 in the development of radioresistance.

The EGFR/SRC/STAT3/HK2 axis identified in this study represents a promising biomarker signature for stratifying TNBC patients who may benefit from radiosensitizing strategies. Phosphorylated STAT3 (p-STAT3^Tyr705^) is frequently elevated in TNBC and is associated with poor prognosis, chemoresistance, and CSC-like properties. Its activation often correlates with radioresistance through sustained anti-apoptotic signaling [43]. Similarly, recent studies have identified overexpressed BUB1, a mitotic checkpoint kinase that regulates non-homologous end joining, as a biomarker and therapeutic target for radiosensitization in TNBC. Inhibition of BUB1 sensitizes tumor cells to radiotherapy without affecting normal cells [44]. Our findings suggest that high expression or activation of pathway components such as p-SRC, p-STAT3, or HK2 could identify tumors that rely on CSC-driven radioresistance. This could guide biomarker-driven selection of SRC/STAT3 inhibitors, such as dasatinib, or EGFR inhibitors in combination with radiotherapy. Such strategies support the growing emphasis on companion diagnostics for signaling vulnerabilities in oncology and have the potential to advance personalized therapies by targeting CSC-specific mechanisms of resistance.

Recent findings indicate that hypoxic conditions and metabolic stress can further amplify SRC and STAT3 activation, establishing a self-reinforcing feedback loop that exacerbates radioresistance in cancer cells [45]. Disrupting this pathological circuit may therefore represent a promising therapeutic strategy to overcome treatment resistance. Collectively, our study advances the current understanding of the non-canonical roles of metabolic enzymes, particularly HK2, and underscores the intricate interplay between growth factor signaling, metabolic reprogramming, and cancer stemness in radioresistant TNBC. These insights provide a strong rationale for dual-targeted therapeutic strategies that combine inhibition of SRC or STAT3 with disruption of HK2 function or upstream EGFR signaling. Such approaches could more effectively target the molecular mechanisms driving therapy resistance and potentially improve clinical outcomes for TNBC patients.

This study has some limitations that have to be acknowledged. The primary limitation of this study is that the findings are based largely on *in vitro* experiments using established TNBC cell lines (MDA-MB-231-derived 231RR and Hs578T). While these models effectively capture key features of radioresistance and CSC activity, such as enhanced tumorsphere formation and pathway-dependent HK2 upregulation, they do not fully replicate the complexity of the tumor microenvironment, immune interactions, or tumor heterogeneity observed in patients. Although our data suggest a predominantly non-metabolic role for HK2 in maintaining CSC properties, with minimal impact on overall glycolytic flux, future studies could examine the nuclear localization of HK2 in radioresistant TNBC cells using subcellular fractionation. Nuclear HK2 has been reported in other cancers to sustain stemness independently of glycolysis [21]. Similarly, while tumorsphere formation provided a robust measure of CSC activity, complementary assays, such as aldehyde dehydrogenase activity or *in vivo* limiting dilution assays, could further validate these findings.

Mechanistically, we did not assess direct transcriptional regulation of HK2, such as STAT3 binding to the HK2 promoter via ChIP. However, previous work by Jiang et al. has shown that STAT3 can directly bind with the HK2 promoter to enhance transcription in breast cancer cells [46], suggesting a potential mechanism to explore in future studies. Future investigations should include *in vivo* validation using patient-derived xenografts or orthotopic models, along with clinical correlation analyses, to confirm the therapeutic potential of targeting the EGFR/SRC/STAT3/HK2 axis for overcoming radioresistance in TNBC.

## 5 Conclusion

**Figure 8:**
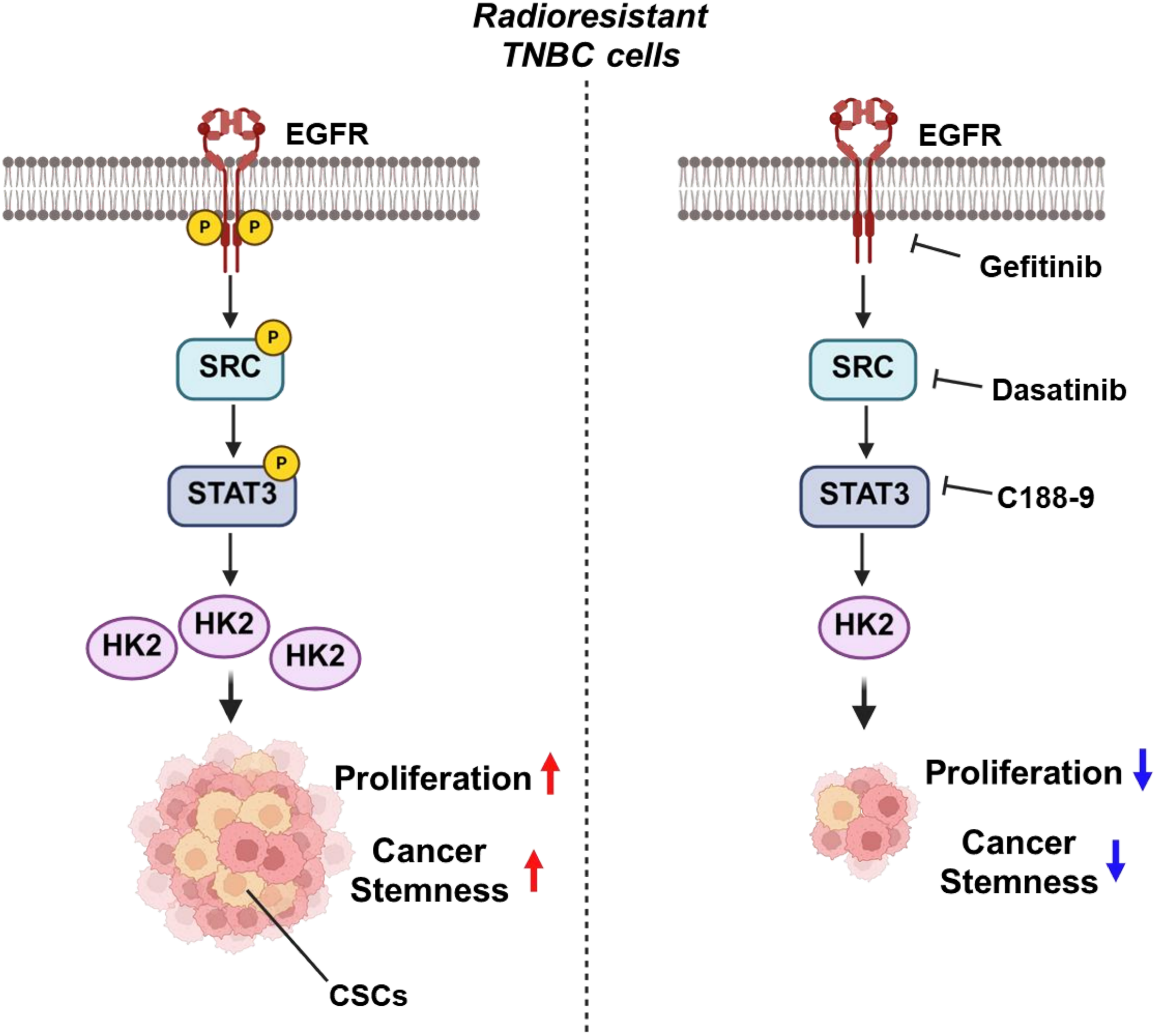
Proposed model of the EGFR–SRC/STAT3–HK2 signaling axis in TNBC radioresistance. Radioresistant TNBC cells exhibit increased activation of EGFR, SRC and STAT3. This activates HK2, promoting cancer stemness and resistance to radiation. HK2 sustains stemness through non-glycolytic mechanisms. Pharmacological inhibition of this pathway (using Gefitinib, Dasatinib or C188-9) reduces tumorsphere formation and downregulates pluripotency factors. Targeting the EGFR–SRC/STAT3–HK2 axis in combination represents a potential strategy to overcome radioresistance and eliminate cancer stem cells in TNBC.

Our study identifies a novel EGFR/SRC/STAT3–HK2 signaling axis that drives CSC properties and radioresistance in TNBC. Radioresistant TNBC cells exhibit increased activation of EGFR, SRC, and STAT3, leading to HK2 upregulation and enhanced CSC proliferation (Fig. 8, left panel). HK2 appears to sustain stemness through non-metabolic mechanisms. Disruption of this pathway, using pharmacological inhibitors (Gefitinib, Dasatinib, C188-9) or genetic approaches, reduces tumorsphere formation, suppresses pluripotency factors, and increases radiation-induced DNA damage (Fig. 8, right panel). Overall, these findings suggest that targeting the EGFR/SRC/STAT3/HK2 axis could overcome radioresistance. However, further validation through *in vivo* models and clinical studies is necessary to substantiate these results.

## Supplementary Materials





## Data Availability

The data used to support the findings of the present study are available from the corresponding authors upon request.

## Supplementary Materials

The supplementary material is available online at https://www.techscience.com/doi/10.32604/or.2026.075190/s1. Fig. S1. Inhibition of SRC proto-oncogene (SRC)/the signal transducer and activator of transcription 3 (STAT3) enhances radiation-induced DNA damage in radioresistant triple negative breast cancer (TNBC) cells. (A) Parental (P) and radioresistant (RR) MDA-MB-231 cells were pretreated with dasatinib or C188-9 for 24 h, followed by exposure to 6 Gy of ionizing radiation. SRC and STAT3 phosphorylation, as well as γH2AX expression, were analyzed by Western blot. GAPDH was used as a loading control. Molecular weights are indicated in kDa. (B) SRC and STAT3 activation was examined by western blot in parental MDA-MB-231 (231-P) cells transduced with lentiviral vectors carrying either an empty control (VC) or a constitutively active SRC mutant (SRC^Y530F^). GAPDH was used as a loading control. Molecular weights are indicated in kDa. (C) Parental MDA-MB-231 (231-P) cells transduced with lentiviral vectors carrying either an empty control (VC) or a constitutively active SRC mutant (SRC^Y530F^), with or without exposure to 6 Gy of ionizing radiation. γH2AX expression was evaluated by Western blot analysis after 30 min. Tubulin was used as a loading control. Molecular weights are indicated in kDa. (D and E) γH2AX expression in 231RR cells treated with dasatinib or C188-9 for 24 h was assessed by immunofluorescence staining (D). The fluorescence intensity of γH2AX foci was quantified using ImageJ software (E). Scale bars represent 100 µm in length. *** *p* < 0.001. Fig. S2. Elevated SRC expression is associated with enhanced glycolytic activity in TCGA breast cancer specimens. (A) A schematic diagram summarizing the Gene Set Enrichment Analysis (GSEA) workflow performed on RNA sequencing (RNA-seq) data from TCGA breast cancer (BRCA) specimens. These were divided into high and low SRC expression groups based on the median SRC mRNA level. (B) The GSEA plots of the glycolysis pathways (HALLMARK_GLYCOLYSIS, left panel; REACTOME_GLYCOLYSIS, right panel). FDR: false discovery rate; NES: normalized enrichment score.
