# Supplementary figures and images for: Targeting SRC/STAT3 Signaling Impairs Cancer Stem Cell Activity by Downregulation of Hexokinase-2 in Radioresistant Triple-Negative Breast Cancer Cells
Source: Oncol Res. 2026 Apr 22;34(5):25. doi: 10.32604/or.2026.075190 (PMC13126418; doi:10.32604/or.2026.075190)

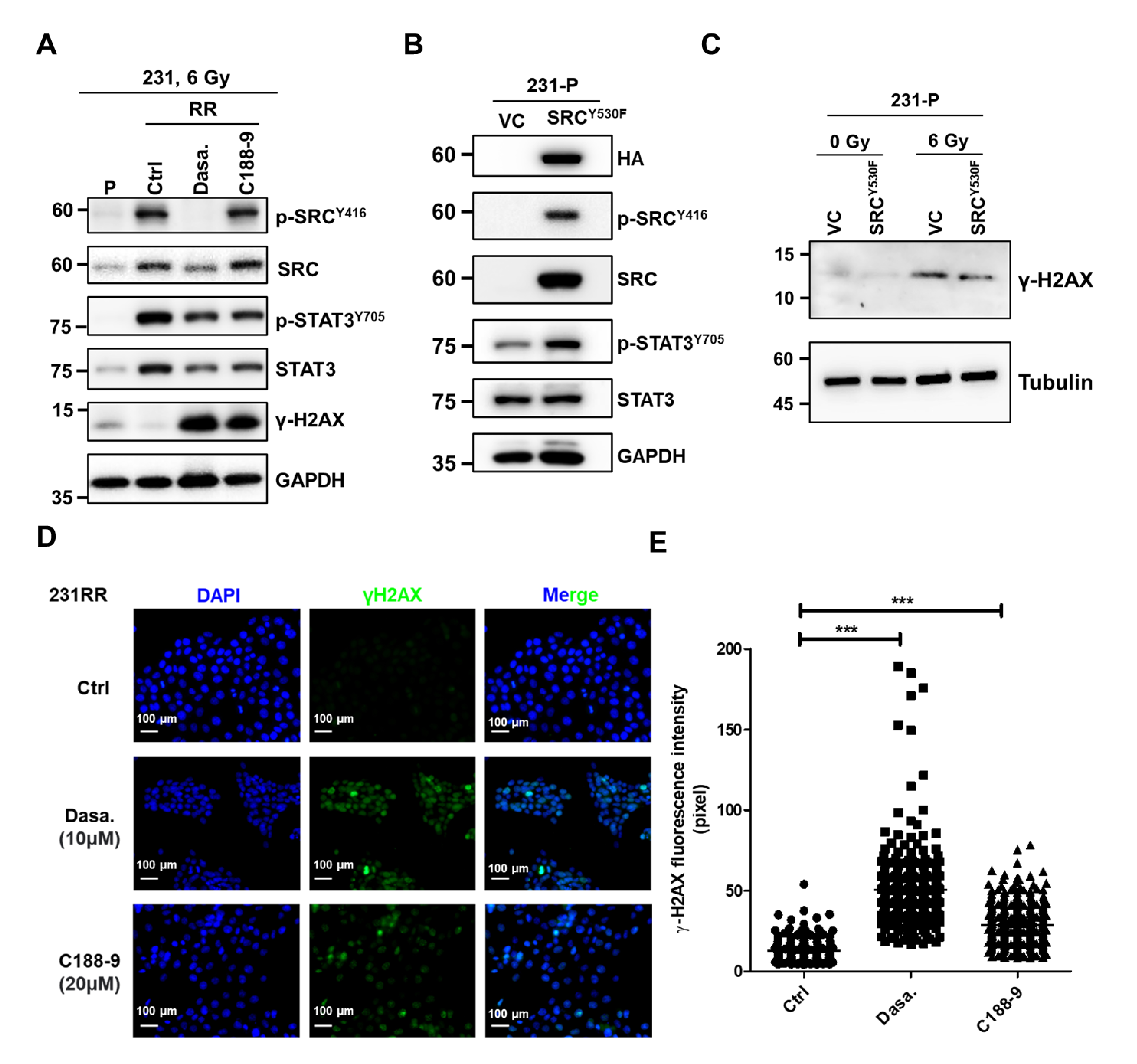

Supplement: Supplementary file 1 [file OncolRes-34-75190-s001.tif]

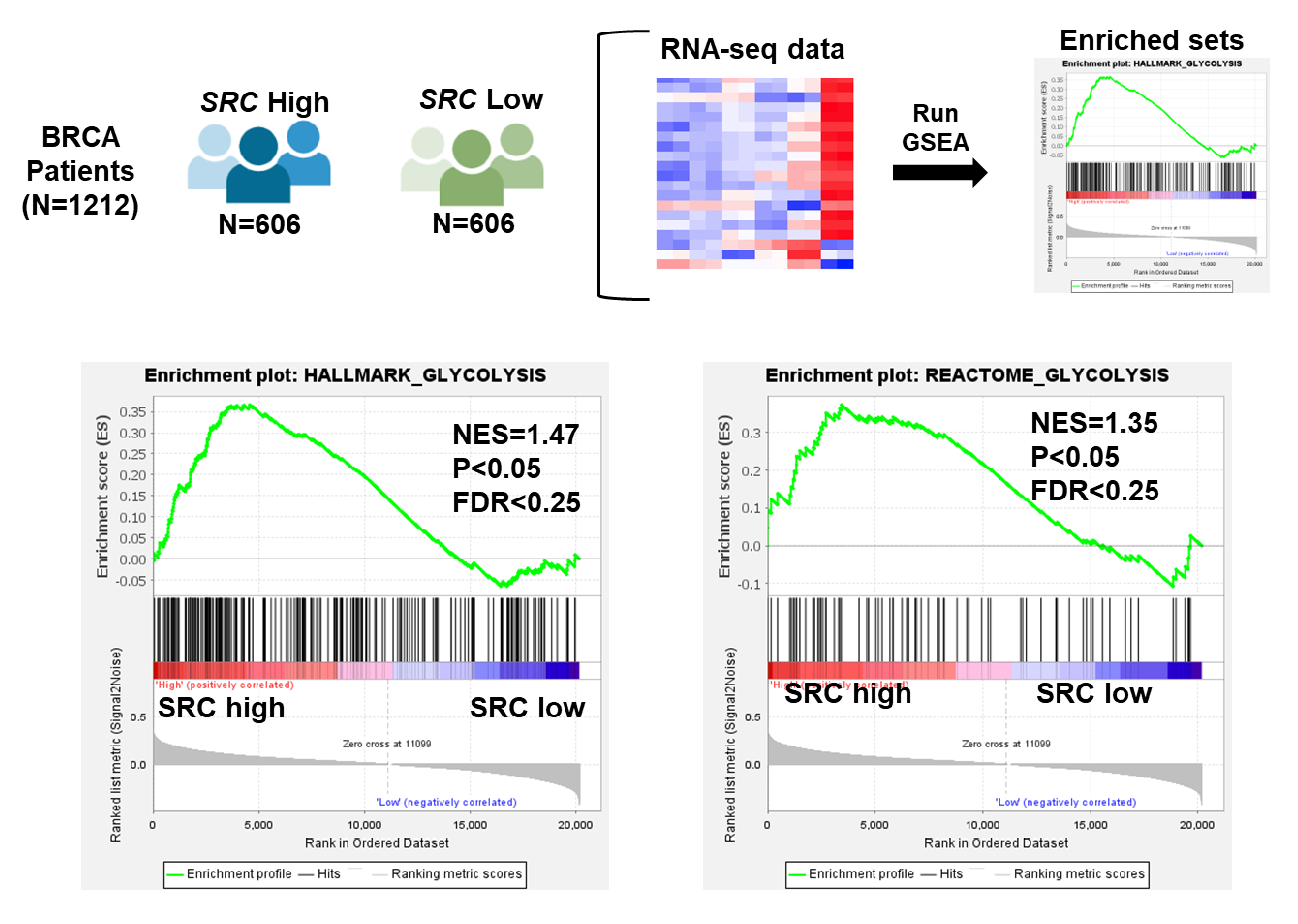

Supplement: Supplementary file 2 [file OncolRes-34-75190-s002.tif]
